# Super-resolution structured illumination microscopy: past, present and future

**DOI:** 10.1098/rsta.2020.0143

**Published:** 2021-06-14

**Authors:** Kirti Prakash, Benedict Diederich, Stefanie Reichelt, Rainer Heintzmann, Lothar Schermelleh

**Affiliations:** ^1^ National Physical Laboratory, TW11 0LW Teddington, UK; ^2^ Department of Paediatrics, Wellcome-MRC Cambridge Stem Cell Institute, University of Cambridge, Cambridge, UK; ^3^ Leibniz Institute of Photonic Technology, Albert-Einstein-Straße 9, 07745 Jena, Germany; ^4^ Institute of Physical Chemistry and Abbe Center of Photonics, Friedrich-Schiller-University, Helmholtzweg 4, Jena, Germany; ^5^ CRUK Cambridge Research Institute, Robinson Way, Cambridge CB2 0RE, UK; ^6^ Faculty of Physics and Astronomy, Friedrich-Schiller-University, Jena, Germany; ^7^ Micron Advanced Bioimaging Unit, Department of Biochemistry, University of Oxford, Oxford OX1 3QU, UK

**Keywords:** super-resolution microscopy, structured illumination microscopy, computational imaging, frugal microscopy, spatial resolution, image processing

## Abstract

Structured illumination microscopy (SIM) has emerged as an essential technique for three-dimensional (3D) and live-cell super-resolution imaging. However, to date, there has not been a dedicated workshop or journal issue covering the various aspects of SIM, from bespoke hardware and software development and the use of commercial instruments to biological applications. This special issue aims to recap recent developments as well as outline future trends. In addition to SIM, we cover related topics such as complementary super-resolution microscopy techniques, computational imaging, visualization and image processing methods.

This article is part of the Theo Murphy meeting issue ‘Super-resolution structured illumination microscopy (part 1)’.

## 1. Introduction

Fluorescence light microscopy is a core technique in life sciences that has contributed to countless major discoveries. However, the diffraction limit, first described by Ernst Abbe in 1873 [1], has restricted the optical resolution to about half the wavelength of the light used, i.e. 200–300 nm in the lateral directions (*x* and *y*), and to about the wavelength of light, i.e. 500–800 nm along the optical axis (*z*). In the past two decades, several super-resolution microscopy (SRM) approaches have been developed that overcome this barrier and push the spatial resolution to the 10–150 nm range, thus closing the gap to electron microscopy [2–5]. These SRM techniques can be further divided into single-molecule localization microscopy (SMLM) [6] that includes techniques like photoactivatable localization microscopy (PALM) [7,8] and stochastic optical reconstruction microscopy (STORM) [9], stimulated emission depletion (STED) [10–12] microscopy, and structured illumination microscopy (SIM) [13,14]. In recognition of these breakthrough developments Eric Betzig, Stefan Hell and William E. Moerner were awarded the Nobel prize for Chemistry in 2014 [15].

Recent SRM developments have focused on combining localization-based microscopy with modulated illumination to push the resolution (precision) towards a few nanometres as in MINFLUX and SIMFLUX [16–19]. However, an increase in resolution requires an increase in local light dosage, which increases photobleaching/phototoxicity (SIM, STED, SMLM) or requires the need to introduce spatial and temporal sparsity (MINFLUX) making imaging comparably slow. Moreover, most SRM techniques mentioned above rely primarily on chemically fixed (i.e. dead) cells, are restricted in the imaged sample volume (two dimensions, single plane, small field-of-views) or are restricted to imaging sparse single entities such as vesicles or single molecules.

**Figure 1.**
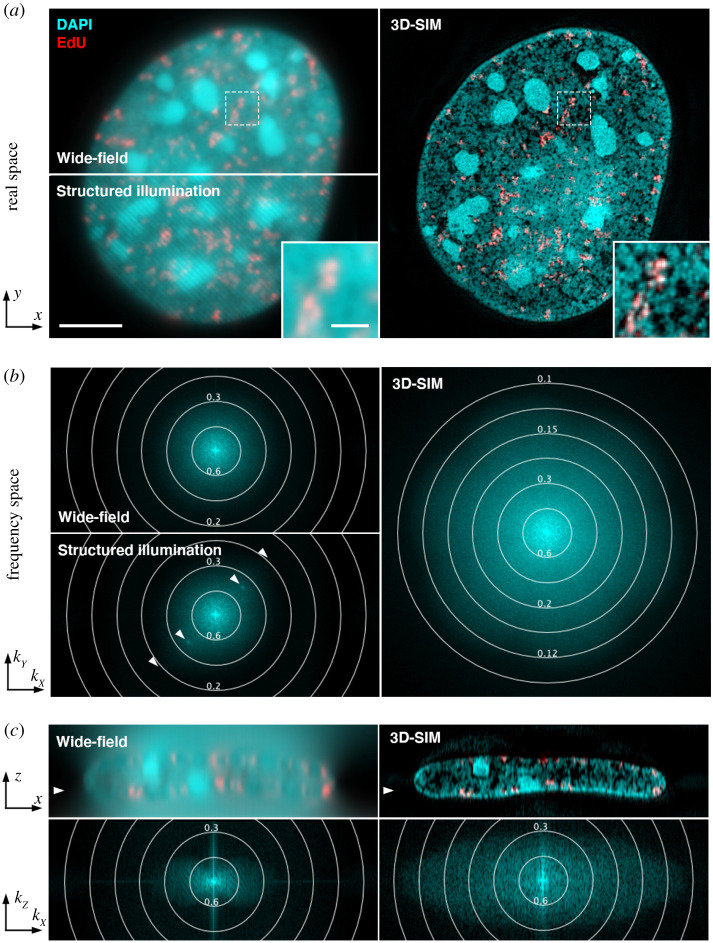
Biological super-resolution imaging with 3D-SIM. (*a*) Mouse C127 mammary epithelial cell nucleus replication labelled with 5-ethenyl-2′-deoxyuridine (EdU, red) for 15 min before fixation with formaldehyde. The thymidine analogue EdU is incorporated into newly synthesized DNA of S-phase cells (here mid-to-late S phase) and detected via click-chemistry with Alexa Fluor 594 azide. DNA is labelled with 4′, 6-diamidino-2-phenylindole (DAPI, cyan). Single *z*-section of an image stack is shown with conventional wide-field illumination (top, left), structured illumination (1 of 15 raw images acquired per *z*-plane with laterally shifted and rotated stripes; bottom, left), and after 3D-SIM reconstruction (right). Note that 3D-SIM resolves higher-order domain organization of chromatin and DNA-free interchromatin regions (inset), as well as the location of nuclear pores in the peripheral chromatin layer visible as DAPI void dots in the central region of the nucleus. Scale bar: 5 and 1 *μ*m (inset). (*b*) Corresponding frequency distribution of the DAPI signal in Fourier (reciprocal) space. Concentric rings indicate the respective spatial resolution in *μ*m. Spots in the raw SI frequency plot (arrowheads) correspond to first- and second-order stripes in the image (generated by a three-beam interference; only coarse first-order stripes are visible in the image). (*c*) Orthogonal cross-section and corresponding frequency distribution of the same dataset. The arrowheads indicate the position of the *z*-section shown in (*a*). Note the twofold extended frequency distribution in the reconstructed data in both lateral and axial direction, that includes the filling-in of the missing frequencies along the *z*-axis of the wide-field frequency plot. (Online version in colour.)

Super-resolution linear SIM is a notable exception [20]. By making use of frequency mixing when exciting samples with a patterned illumination followed by computational unmixing and reconstruction, SIM achieves a twofold resolution increase over conventional diffraction-limited fluorescence microscopy in two dimensions or three dimensions (figure 1). While the numerical resolution improvement is moderate compared to other SRM techniques, it pushes SIM into an application sweet spot with many macromolecular structures and their range of motion falling in the size range of 100–300 nm [21–23]. By not asking for the highest spatial resolution, SIM is less demanding in terms of photon budget and therefore more compatible with live-cell imaging [24–29]. In addition, it offers high contrast volumetric imaging with relatively large field-of-views and comparably high temporal resolution which makes it suitable for high-content and live-cell imaging [30–32]. Taken together, the uniquely balanced combination of properties has made SIM remarkably successful for a wide range of biological applications promoting new discoveries [22,33–48].

## 2. Recent and future developments in SIM

SIM has been available in commercial instruments for more than 10 years and has been successfully established in many laboratories and core facilities around the world. However, wider dissemination of SIM has been curtailed by the complexity of instruments and its proneness to reconstruction artefacts when sample properties, system calibration and parameter settings are not carefully matched [49]. Thus, to lower the activation energy for research labs to venture into super-resolution SIM, many recent developments aim at further performance/resolution enhancement and ‘democratizing’ SIM by

— using nonlinear SIM approaches [50–53] or by combinations with single-molecule imaging [16,17,54–56],
— exploiting correlative and combinatorial fluorescence imaging approaches with multifocal [57,58], 2-photon [59–61] and light-sheet microscopy [62,63],
— implementing the technique into smaller and more cost-efficient set-ups [64–66],
— making the method more robust against artefacts using alternative illumination schemes [67–69] and/or intelligent data processing [70,71],
— increasing its application range, e.g. by the implementation of adaptive optics [72–74], and cryo-imaging [47,75].

Important advancements are being made to improve and simplify the instrumentation. For instance, newly designed optical set-ups using an open-sources toolbox [65] or waveguide-based photonic chips [70], and open-source software development [76] will reduce the cost of SR imaging by enabling SIM on smaller and more affordable devices. Moreover, next-generation sCMOS cameras offer increased frame rates of up to 500 fps and higher sensitivity with reduced noise levels to enable faster high-quality SIM imaging for observing cellular dynamics [77–79]. For maximal imaging speed, rolling shutter schemes can be combined with SIM data acquisition [28,80].

In parallel, the rapidly evolving field of computational microscopy is paving the way to circumvent the resolution limit without the necessity of specialized hardware reducing the complexity of instrumentation. For example, machine learning (ML) assisted denoising has been demonstrated to improve SIM reconstructions by reducing artifacts and boosting the effective resolution for 2D-SIM [65,66,70,71,81–84]. Thus, by combining physical data acquisition with smart image processing algorithms, previously inaccessible information can be recovered. At the same time, this brings up new problems of validation of the results from ML methods, therefore calling for new standards on the performance of these algorithms. Finally, adaptive optics (AO) implementations have demonstrated great potential to correct sample aberrations and restore performance when imaging challenging samples [72–74]. However, AO does not solve the problem of reduced illumination pattern contrast in the presence of significant out-of-focus background. Thus, combinations of AO-SIM with light sheet illumination, 2-photon or photoswitching methods [57–63] present avenues for next developments.

**Frequently asked questions in super-resolution structured illumination microscopy field**

The following questions have not always been agreed upon in the super-resolution/SIM field, and with this special issue, we hope to address some of these:

1. What is SRM and should diffraction-limited linear SIM be classified as ‘super-resolution’?
2. Should High-NA TIRF-SIM, which can achieve a lateral resolution of down to 84 nm, be considered as diffraction limited?
3. Can nonlinear SIM become broadly applicable and live-cell compatible?
4. Do you need ‘switching’ of states for nonlinear super-resolution?
5. How can information about single-molecule detection be best combined with the knowledge of the illumination structure?
6. Do high-quality SIM images require reconstruction in Fourier space?
7. Can SIM be used for deep tissue imaging?
8. How can the fundamental limitation of SIM, i.e. generating sufficient stripe contrast in densely labelled and/or extended biological structures due to out-of-focus light, be addressed?
9. Should image scanning microscopy be considered a form of SIM and what forms of structured illumination could be used other than stripes?
10. Can SIM be used to improve the resolution of (Rayleigh scattering) transmission microscopy?
11. How does sparse illumination compare to dense illumination in linear and nonlinear SIM?
12. Can we generate ‘true’ super-resolution images from simple instruments enhanced with machine-learning-based algorithms?
13. Can research-grade super-resolution (SIM) microscopes be built cost-efficiently?

For answers to these fundamental questions in super-resolution microscopy, please refer to [85].

## 3. The wider-reaching social implications

SRM has evolved into a highly interdisciplinary field requiring experts from physics, engineering, chemistry, biology and computer sciences driving innovations to further increase spatial and temporal resolution and ultimately biological applicability. The global advances in SRM need to be synchronized to inspire researchers in the field of microscopy to combine their methods with techniques from different areas.

SRM and SIM, in particular, have become important tools of basic science discovery. However, there are still considerable activation barriers for researchers to venture into SRM, as instruments are not yet ‘turn-key’, and due to the relatively steep learning curve required to use SIM (or any other SRM method) to its full potential. Furthermore, the application of SRM to study human pathologies and for medical diagnosis is still in its infancy, but will foreseeably play a significant role in the future. New SIM modalities, implementation of AO and machine-learning algorithms will help to democratize SIM and increase its usage in biomedical research.

Super-resolution microscopy is very often an expensive undertaking, thus limiting its broader application. In this meeting, we plan to kick-off the idea of an open-source ‘openSIM’ system inspired by its derivatives in the light-sheet community. Open discussions where ideas and requirements for a prototype are gathered are a first and significant step to make science not only affordable but available. This special issue shows current progress in SIM development and application.

## Data accessibility

This article has no additional data.
